# Optimization of Liquid Culture of Extracellular Flavonoids of *Sanghuangporus* and Evaluation of Antioxidant Activity of Fermentation Broth

**DOI:** 10.3390/foods15030455

**Published:** 2026-01-28

**Authors:** Yingbai Wang, Junliang Chen, Yingkun Yang, Zhaojuan Zhang, Weiming Cai, Xingru Yan, Yu Peng, Yu Li, Pu Liu

**Affiliations:** 1 College of Plant Protection, College of Mycology, Jilin Agricultural University, Changchun 130118, China; 15526943119@163.com (Y.W.); 17843098488@163.com (Z.Z.); yanxingru2022@163.com (X.Y.); pengyu202205@163.com (Y.P.); 2 Engineering Research Center of Chinese Ministry of Education for Edible and Medicinal Fungi, Jilin Agricultural University, Changchun 130118, China; 3 Edible Fungus Industry Center of Qingyuan County (Edible Fungus Research Center of Qingyuan County), Lishui 323800, China; chenjunliang1026@126.com (J.C.); yangyingkun2020@163.com (Y.Y.); 4 Institute of Horticulture, Zhejiang Academy of Agricultural Sciences, Hangzhou 310021, China; caiwm527@126.com

**Keywords:** *Sanghuangporus*, response surface analysis, extracellular flavonoids, fermentation broth, antioxidant

## Abstract

Species of *Sanghuangporus* produce abundant bioactive compounds, including flavonoids, polysaccharides, and triterpenoids, among which flavonoids exhibit prominent antioxidant activity and great development potential. Taking the peak extracellular flavonoid (EF) concentration in fermentation broth as the index, we optimized the liquid fermentation media and conditions for three *Sanghuangporus* strains (wild *S. vaninii* Z-0090, cultivar *S. vaninii* Z-0119, and cultivar *S. baumii* Z-0118) via single-factor experiments and the Box–Behnken response surface methodology. We further evaluated the antioxidant activity of the fermentation broth and analyzed its correlation with EF concentration using Pearson correlation analysis. After optimization, the EF concentrations of strains Z-0090, Z-0119 and Z-0118 were increased by 22.82%, 13.47% and 16.66%, respectively, compared with the control group. Antioxidant assays showed that strain Z-0090 had the highest hydroxyl radical scavenging rate (88.63%), strain Z-0119 presented balanced performance across all antioxidant indicators, and strain Z-0118 exhibited the strongest ABTS radical scavenging capacity (184.96 μg Trolox equivalents/mL), which was highly correlated with its EF concentration. This study provides a theoretical basis for the application of *Sanghuangporus* EFs in food, medicine, and industrial flavonoid production.

## 1. Introduction

Excessive production of reactive oxygen species (ROS) can damage cells and organs, thereby contributing to the development and progression of chronic diseases [1]. ROS-induced damage to DNA, proteins, and lipids disrupts cellular integrity, underscoring the urgent need for safe and effective antioxidants [2]. Recently, researchers have extensively studied morin flavonoids for their exceptional free radical scavenging capabilities, as well as their lipid-lowering and hypoglycemic activities [3].

*Sanghuangporus*, a member of the family Hymenochaetaceae, is a medicinal fungus long utilized in traditional Chinese medicine and known for its wealth of bioactive metabolites, such as flavonoids, terpenoids, and steroids [4]. The various compounds contained in mulberry yellow have been proven by modern medical technology to have excellent anti-inflammatory, anti-tumor, and immunomodulatory effects. In addition, they also have the ability to improve health, including lowering blood sugar, improving sleep, anti-aging, and protecting the nerves [5]. Flavonoids extracted from *Sanghuangporus* have been confirmed to be one of the core contributors to its antioxidant activity, showing significant scavenging effects on 2,2-Diphenyl-1-picrylhydrazyl (DPPH) radicals, 2,2′-Azinobis(3-ethylbenzothiazoline-6-sulfonic acid) (ABTS) radicals and hydroxyl radicals [6]. A comparative study conducted in 2023 on 15 strains of *Sanghuangporus* in terms of scavenging multiple free radicals and antioxidant capacity indicated that flavonoids and ascorbic acid contributed the most to the antioxidant effect, followed by polyphenols and triterpenoids, while polysaccharides had the weakest antioxidant effect [7]. Importantly, extracellular flavonoids (EFs), secreted into the culture medium during liquid fermentation, offer distinct advantages over intracellular flavonoids. These benefits include easier extraction processes, lower purification costs, and the feasibility of industrial-scale production [8]. Consequently, the extracellular flavonoids of *Sanghuangporus* present a promising resource for developing natural antioxidants.

Despite these advantages, research on the extracellular flavonoids of *Sanghuangporus* remains fragmented. Most studies have focused on intracellular flavonoids in the fruiting bodies or mycelia, with limited attention to their extracellular counterparts. The yield of extracellular flavonoids from liquid fermentation is significantly influenced by culture conditions, including the carbon source, nitrogen source, and pH. Unoptimized processes lead to low yields, creating a bottleneck that restricts practical applications. Few studies have addressed extracellular flavonoids of *Sanghuangporus*. Simple optimizations to enhance yield were made by Zhao et al. [9], while Li et al. reported antioxidant activity in the fermentation broth without linking it to specific active components [10]. Consequently, research is still lacking on how optimizing liquid culture conditions affects the biosynthesis of extracellular flavonoids and their antioxidant efficacy. In addition, in terms of optimizing techniques, the selection of the response surface method (RSM) may be of great significance, as it requires fewer experiments compared to the single-factor method [11]. RSM is widely used in the process optimization of fermentation. This technology can identify the interaction between different variables by establishing appropriate digital models.

To advance research in this field, this study systematically optimized the liquid culture parameters for extracellular flavonoids from *Sanghuangporus* using response surface methodology. In addition, it assessed the dynamic changes in the antioxidant activity of the fermentation broth. The objective was to establish a viable strategy to enhance the yield of extracellular flavonoids and offer a theoretical foundation for their industrial application as natural antioxidants.

## 2. Materials and Methods

### 2.1. Test Strains of Sanghuangporus

The specimen of *Sanghuangporus vaninii* was collected from Jinzifeng National Forest Park in Qingyuan County and its wild strain Z-0090 was obtained through tissue isolation and purification. It is currently preserved at the Edible Fungus Industry Center of Qingyuan (Edible Fungus Research Center of Qingyuan County). The *Sanghuangporus vaninii* cultivar (strain Z-0119) is from the Zhejiang Academy of Agricultural Sciences, and the *Sanghuangporus baumii* cultivar (strain Z-0118) is from the Shanghai Academy of Agricultural Sciences.

### 2.2. Strain Activation

The *Sanghuangporus* strains were inoculated onto Potato-Dextrose-Agar (PDA) solid medium plates (composed of 200 g peeled potatoes, 20 g glucose, 20 g agar, 1000 mL distilled water, with pH natural) and cultured in a constant-temperature incubator at 30 °C for 10 d.

### 2.3. Cultivation of Primary Seed Liquid

Six pieces of *Sanghuangporus* fungus mycelia with a diameter of 5 mm were taken from the plate medium using a puncher and inoculated into the basal medium (the liquid volume in a 250 mL Erlenmeyer flask was 80%). The flask was then placed in a constant-temperature shaking incubator at 26 °C and 180 rpm for 10 days to obtain the seed liquid.

### 2.4. Secondary Seed Liquid Culture

Liquid fermentation media with varied ingredient ratios were prepared according to the experimental design. The mycelia of the well-cultured primary seed liquid were disrupted using a homogenizer, and 20 mL of the treated seed liquid was inoculated into 200 mL of the liquid fermentation medium. Cultivation was carried out at 26 °C with shaking at 180 rpm, and fermentation was terminated on the 10th day.

### 2.5. Detection of Extracellular Flavonoid Concentration

The standard curve was constructed as follows. The rutin standard stock solution (1 mg/mL) was prepared by weighing 2 mg of the rutin standard substance into a clean Eppendorf (EP) tube, adding 2 mL of 60% ethanol extraction solution, and vortexing thoroughly for complete dissolution; the stock solution was stored for subsequent use. Six concentration gradients (e.g., 0, 0.2, 0.4, 0.6, 0.8, 1 mg/mL) were prepared by diluting the stock solution with 60% ethanol, and concentrations could be adjusted based on actual sample characteristics. For standard tube preparation, 50 μL of the rutin standard solution and 15 μL of Reagent 1 were mixed, incubated at 25 °C for 6 min, supplemented with 30 μL of Reagent 2, incubated at 25 °C for another 6 min, and then mixed with 105 μL of Reagent 3 followed by incubation at 25 °C for 15 min. The absorbance (Astandard) was measured at 510 nm. A zero-concentration control tube was prepared by substituting 50 μL of 60% ethanol for the rutin standard solution, following the same incubation and reagent addition procedures, and its absorbance (A0) was measured at 510 nm. The corrected absorbance (ΔA) was calculated as ΔA = Astandard − A0. The standard curve was plotted with ΔA on the y-axis and standard concentration on the x-axis, giving the regression equation: y = 1.6283x + 0.0045 (x = standard concentration in mg/mL; y = ΔA).

For extracellular flavonoid concentration determination, 4 mL of the fermentation medium was transferred to a 5 mL centrifuge tube, sealed, and centrifuged at 3500 rpm for 10 min to collect the supernatant. The ΔA value of the supernatant was measured using the same protocol as for the standard curve, and the extracellular flavonoid concentration was calculated by substituting the measured ΔA into the regression equation.

### 2.6. Plotting of the Flavonoid Production Curve

Six plugs of *Sanghuangporus* mycelia with a diameter of 5 mm were punched out and inoculated into 250 mL Erlenmeyer flasks containing 200 mL of basal medium (glucose 30 g/L, yeast extract 5 g/L, MgSO_4_·7H_2_O 1 g/L, KH_2_PO_4_ 1 g/L, Vitamin B_1_ 0.1 g/L). These Erlenmeyer flasks were cultured in a shaker at 26 °C and 180 rpm in the dark for 10 days. The flavonoid concentration of the fermentation broth began to be detected at 48 h after inoculation, and this detection was then carried out every 48 h to determine the time when the extracellular flavonoid concentration reached to the peak period.

### 2.7. Single-Factor Experiment

Single-factor experiments were conducted by altering the types of carbon sources, nitrogen sources, growth factors, and inorganic salts to determine the optimal liquid fermentation medium. These experiments aimed to optimize the nutritional components for the production of active substances. These bioactive substances in the fermentation broth can be used to enhance the health-beneficial effects in drugs or foods.

### 2.8. Single-Factor Experiment of Carbon Sources

By altering the carbon source in the basal medium formula, the effects of different carbon sources on the flavonoid concentration at the peak period in the fermentation broth of *Sanghuangporus* mycelium were analyzed. The following carbon sources were used, such as glucose, lactose, sucrose, rice flour, soluble starch, corn flour, and potato flour, with a concentration of 30 g/L for each carbon source.

### 2.9. Single-Factor Experiment of Nitrogen Sources

By altering the nitrogen source in the basal medium formula, the effects of different nitrogen sources on the peak period concentration of flavonoids in the fermentation broth of *Sanghuangporus* mycelia were analyzed. In each experimental group, beef extract, peptone, yeast powder, potassium nitrate, glutamic acid, urea, and a combination of 10 g/L yeast powder and 5 g/L peptone were used as nitrogen sources, respectively. Each nitrogen source was tested at a concentration of 5 g/L.

### 2.10. Single-Factor Experiments of Inorganic Salts

By altering the types of inorganic salts in the basal medium formula, the effects of different inorganic salts on the flavonoid concentration at the peak stage in the fermentation broth of *Sanghuangporus* mycelia were analyzed. In each experimental group, KH_2_PO_4_, CaCl_2_, MgSO_4_·7H_2_O, ZnSO_4_·7H_2_O, FeSO_4_·7H_2_O, and a mixture of 1 g/L MgSO_4_·7H_2_O and 1.5 g/L KH_2_PO_4_ were used, respectively.

### 2.11. Single-Factor Experiment on the Initial pH of the Fermentation Broth

Based on the initial culture parameters (pH 7.0, inoculum size of 10% *v*/*v*, rotation speed of 180 rpm), and keeping other variables constant, the initial pH of the fermentation broth was independently adjusted to 6.0, 7.0, and 8.0, respectively, to analyze the effects of different initial pH values of the fermentation broth on the flavonoid concentration at the peak period in the *Sanghuangporus* mycelium fermentation broth.

### 2.12. Single-Factor Experiment on Inoculum Volume

Based on the initial culture parameters (pH 7.0, inoculum size 10% *v*/*v*, rotation speed 180 rpm), and keeping other variables constant, the inoculum volumes were independently adjusted to 5% *v*/*v*, 10% *v*/*v*, and 15% *v*/*v*, respectively, to analyze the effects of different inoculum volumes on the concentration of flavonoids at the peak period in the fermentation broth of *Sanghuangporus* mycelia.

### 2.13. Single-Factor Experiment on Rotational Speed

Based on the initial culture parameters (pH 7.0, inoculum volume of 10% *v*/*v*, rotation speed of 180 rpm), and keeping other variables constant, the rotation speed was independently adjusted to 120 rpm, 150 rpm, and 180 rpm, respectively. The influence of different rotation speeds on the concentration of flavonoids at the peak period in the fermentation broth of *Sanghuangporus* mycelia were analyzed.

### 2.14. Response Surface Optimization Experiment Using the Box–Behnken Design

Response surface methodology (RSM) based on the Box–Behnken design (BBD) was applied to optimize the concentrations of carbon sources, nitrogen sources, and inorganic salts. The optimal carbon source, nitrogen source, and inorganic salt (identified in prior screening experiments) were designated as independent variables, and a three-factor, three-level experimental design was implemented by assigning low, medium, and high concentration levels to each variable. The extracellular flavonoid concentration in the fermentation broth was selected as the response variable for the subsequent optimization and statistical analysis.

### 2.15. Determination of the Antioxidant Capacity of the Fermentation Broth

Preparation of fermentation broth samples involved the following steps: each time, take 4 mL of the culture medium in a 5 mL centrifuge tube, seal and centrifuge it at 1200× *g* for 10 min. The supernatant was obtained for detection.

The ABTS free radical scavenging activity kit was purchased from Sangon Biotech (Shanghai) Co., Ltd. (Shanghai, China), and the assay was performed as follows. A microplate reader preheated for more than 30 min was calibrated to a wavelength of 734 nm. Three types of tubes were prepared: a measurement tube containing 10 μL of fermentation broth and 190 μL of working solution, a control tube with 10 μL of fermentation broth and 190 μL of absolute ethanol, and a blank tube consisting of 10 μL of absolute ethanol and 190 μL of working solution. After thorough mixing, all tubes were incubated in the dark at room temperature (25 °C) for 6 min, and the solutions were then sequentially transferred to a 96-well plate for absorbance (A) measurement at 734 nm. The ABTS free radical scavenging rate was calculated using the formula: Scavenging rate (%) = [(1 − (A_measurement − A_control) ÷ A_blank) × 100%]. The ABTS free radical scavenging activity was expressed as μg Trolox/mL, calculated by the equation: Scavenging activity (μg Trolox/mL) = [(scavenging rate + 0.4867) ÷ 0.5167] × D, where Trolox has a molecular weight of 250.29 and D represents the dilution factor (set to 1 if no dilution was performed).

The DPPH radical scavenging activity kit was purchased from Sangon Biotech (Shanghai) Co., Ltd. (Shanghai, China), and the assay was conducted as follows. A microplate reader was preheated to 25 °C for more than 30 min and calibrated to a wavelength of 517 nm. Three types of tubes were prepared: a measurement tube containing 150 μL of fermentation broth and 150 μL of working solution, a control tube with 150 μL of fermentation broth and 150 μL of 80% methanol, and a blank tube consisting of 150 μL of 80% methanol and 150 μL of working solution. After thorough mixing, the tubes were allowed to stand in the dark at room temperature (25 °C) for 30 min, followed by centrifugation at 13,800× *g* for 5 min at room temperature. A 200 μL aliquot of the supernatant was transferred to a 96-well plate, and the absorbance (A) was measured at 517 nm. The DPPH radical scavenging rate was calculated using the formula: Scavenging rate (%) = [(1 − (A_measurement − A_control) ÷ A_blank) × 100%]. The DPPH radical scavenging activity was expressed as μg Trolox equivalents/mL, calculated by the equation: Scavenging activity (μg Trolox equivalents/mL) = [(scavenging rate − 0.7122) ÷ 2.8495 × V_1_] ÷ V_1_ × D. Here, Trolox equivalents have a molecular weight of 250.29, V_1_ denotes the sample volume in the reaction system, and D represents the dilution factor (set to 1 if no dilution was performed).

The hydroxyl radical scavenging activity kit was purchased from Sangon Biotech (Shanghai) Co., Ltd. (Shanghai, China), and the assay was performed as follows. A microplate reader was preheated to 25 °C for more than 30 min and calibrated to a wavelength of 510 nm. Three types of tubes were prepared with the following components: a measurement tube containing 50 μL of Reagent 1, 50 μL of Reagent 2, 50 μL of fermentation broth, 200 μL of distilled water, and 50 μL of Reagent 3; a control tube with 50 μL of Reagent 1, 50 μL of Reagent 2, 50 μL of fermentation broth, and 250 μL of distilled water; and a blank tube consisting of 50 μL of Reagent 1, 50 μL of Reagent 2, 250 μL of distilled water, and 50 μL of Reagent 3. After thorough mixing, the tubes were incubated at 37 °C for a precise 20 min. If turbidity was observed in the measurement or control tube, centrifugation was conducted at 6200× *g* for 5 min at room temperature. A 200 μL aliquot of the clear supernatant was transferred to a 96-well plate, and the absorbance (A) of each tube was read immediately at 510 nm. The hydroxyl radical scavenging rate was calculated using the formula: Scavenging rate (%) = [A_blank − (A_measurement − A_control)] ÷ A_blank × 100%.

The Total Antioxidant Capacity (T-AOC) assay was performed in accordance with the manufacturer’s instructions provided with the kit (Sangon Biotech, Shanghai, China). One unit (U) of T-AOC was defined as the amount of antioxidant activity that induces a 0.01 increase in the absorbance of the reaction system per 1.0 mL of sample within 1 min at 37 °C. The assay principle is based on the ability of antioxidants to reduce Fe^3+^-tripyridine triazine (Fe^3+^-TPTZ) to form a blue Fe^2+^-TPTZ complex under acidic conditions. The total antioxidant capacity of the sample was then determined by measuring the absorbance of the blue Fe^2+^-TPTZ complex at a wavelength of 590 nm.

The superoxide anion scavenging activity kit was purchased from Sangon Biotech (Shanghai) Co., Ltd. (Shanghai, China), and the assay was conducted as follows. A microplate reader was preheated to 25 °C for more than 30 min and calibrated to a wavelength of 320 nm. Both Reagent 1 and Reagent 2 were pre-incubated at room temperature (25 °C) for 20 min prior to use. Three types of tubes were prepared with the following components: a measurement tube containing 200 μL of Reagent 1, 10 μL of fermentation broth, 20 μL of Reagent 2, and 10 μL of Reagent 3; a control tube with 200 μL of Reagent 1, 10 μL of fermentation broth, 20 μL of distilled water, and 10 μL of Reagent 3; and a blank tube consisting of 200 μL of Reagent 1, 10 μL of distilled water, 20 μL of Reagent 2, and 10 μL of Reagent 3. After thorough mixing for 3 min, the absorbance (A) of each tube was measured at 320 nm. The superoxide anion scavenging rate was calculated using the formula: Scavenging rate (%) = [1 − (A_measurement − A_control) ÷ A_blank] × 100%.

### 2.16. Statistical Analysis

The Box–Behnken experiments were designed using Design Expert (ver. 13.0) software. The graphs were obtained using Origin (ver. 2021) software. The correlation between extracellular flavonoids concentrations and the antioxidant activity of the fermentation broth was determined by Pearson coefficient analysis. All experiments were performed in triplicate.

## 3. Results

### 3.1. Determination of the Curve of Extracellular Flavonoid Production

**Figure 1:**
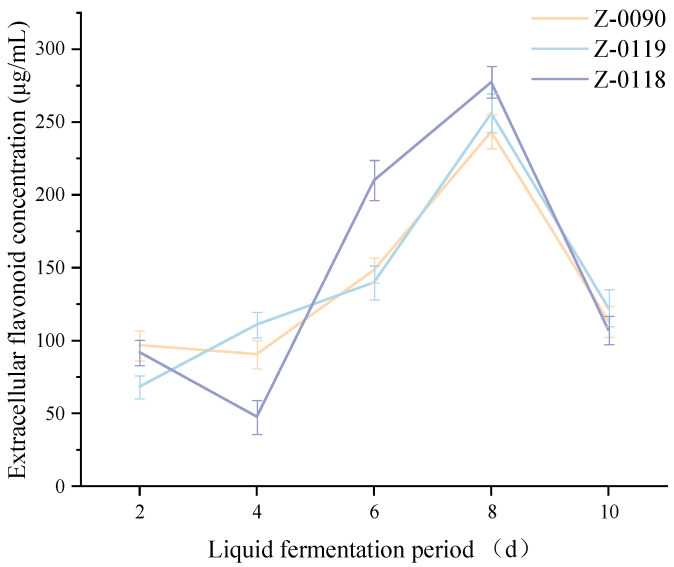
Curve showing the change in extracellular flavonoid concentration in liquid fermentation.

As shown in Figure 1, the extracellular flavonoid concentration in the three *Sanghuangporus* strains increased following the progressed fermentation days, and the peaking values for these three strains were all on the 8th day before declining. A comparison of the peak flavonoid values revealed that strain Z-0118 had a significantly higher concentration than the other two strains, while strains Z-0090 and Z-0119 showed no significant difference.

### 3.2. Effects of Carbon Source Types on the Concentration of Extracellular Flavonoids in Sanghuangporus

**Figure 2:**
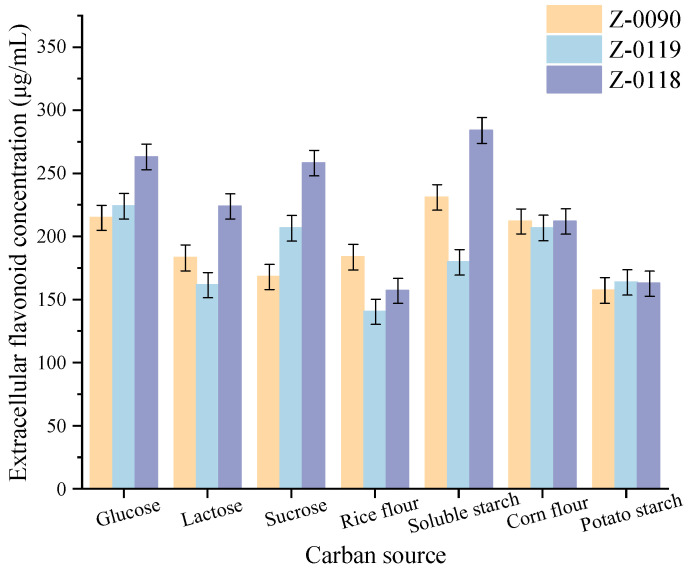
Effects of different carbon sources on the extracellular flavonoid concentration of *Sanghuangporus*.

Figure 2 showed that soluble starch enhanced the extracellular flavonoid concentration in *Sanghuangporus* strains Z-0090 and Z-0118, whereas glucose increased the flavonoid concentration in strain Z-0119. These findings suggested that soluble starch was the optimal carbon source for strains Z-0090 and Z-0118, while glucose was suitable for strain Z-0119. Under liquid fermentation with these optimal carbon sources, the highest extracellular flavonoid concentrations were 231.19 μg/mL for strain Z-0090, 207.03 μg/mL for strain Z-0119, and 284.12 μg/mL for strain Z-0118.

### 3.3. Effects of Nitrogen Source Types on the Extracellular Flavonoid Concentration of Sanghuangporus

**Figure 3:**
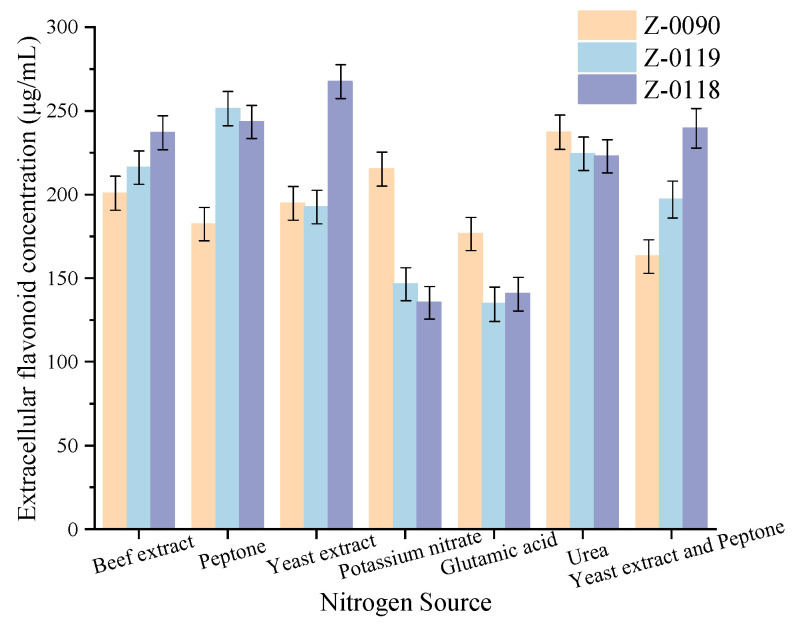
Effects of different nitrogen sources on the extracellular flavonoid concentration of *Sanghuangporus*.

Figure 3 showed that urea significantly enhanced the extracellular flavonoid concentration in strain Z-0090 and slightly in strain Z-0119. Peptone was effective in increasing the extracellular flavonoid concentration of strain Z-0119, while yeast powder increased that of strain Z-0118. These findings indicated that urea was the most effective nitrogen source for strain Z-0090, peptone for strain Z-0119, and yeast powder for strain Z-0118. Liquid cultures utilizing these optimal nitrogen sources, the extracellular flavonoid concentrations for strains Z-0090, Z-0119, and Z-0118 reached 237.37 μg/mL, 251.49 μg/mL, and 267.55 μg/mL, respectively.

### 3.4. Effects of Inorganic Salt Types on the Extracellular Flavonoid Concentration of Sanghuangporus

**Figure 4:**
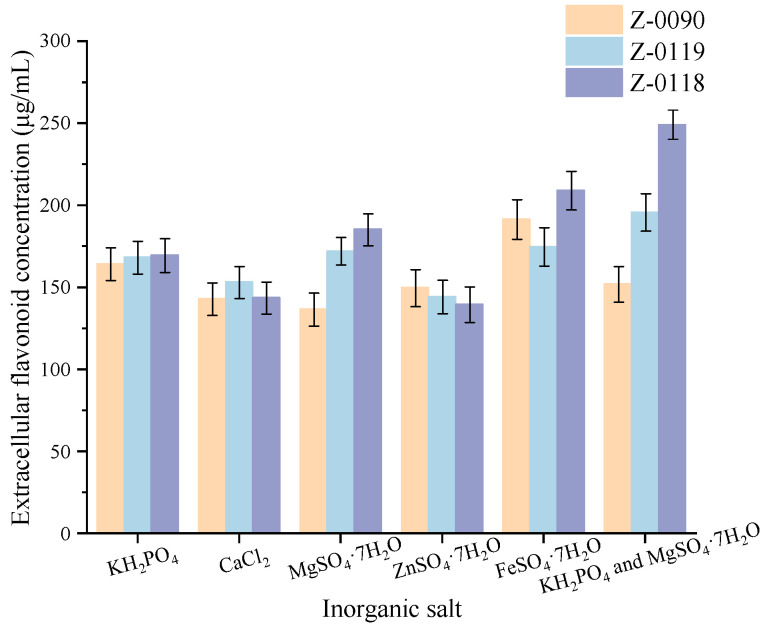
Effects of different inorganic salts on the extracellular flavonoid concentration of *Sanghuangporus*.

Figure 4 showed that using KH_2_PO_4_ and MgSO_4_·7H_2_O as the inorganic salts resulted in the highest extracellular flavonoid concentrations for strains Z-0119 and Z-0118, measuring 195.78 μg/mL and 249.17 μg/mL, respectively. In contrast, FeSO_4_·H_2_O proved optimal for strain Z-0090, achieving an extracellular flavonoid concentration of 191.56 μg/mL in liquid culture.

### 3.5. Optimization of Cultivation Conditions

**Figure 5:**
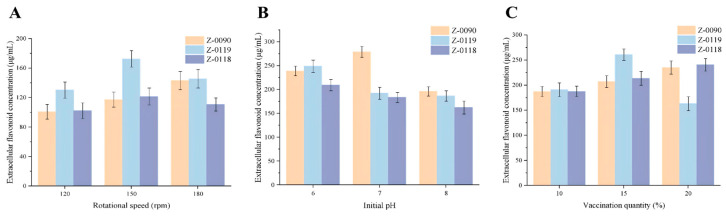
Effects of different cultivation conditions on the extracellular flavonoid concentration of *Sanghuangporus*. (**A**–**C**) Effects of different cultivation conditions on the extracellular flavonoid concentrations of strains (**A**) Rotational speed, (**B**) Initial pH, and (**C**) Vaccination quantity.

The shaking speed primarily influenced the oxygen concentration in the liquid medium. The results indicated that strain Z-0090 reached the highest extracellular flavonoid concentration at a rotation speed of 180 rpm, whereas attains Z-0119 and Z-0118 reached their peak extracellular flavonoid concentrations at a shaking speed of 150 rpm. This suggested that a lower shaking speed was detrimental to the extracellular flavonoid yield during the liquid fermentation of *Sanghuangporus* (Figure 5A), which might because the reduced shaking speed did not facilitate adequate contact of nutrients between the liquid and the mycelium.

The pH environment significantly affected mycelial growth and metabolite synthesis. An optimal pH could enhance the metabolic activities of mycelia. As shown in Figure 5B, this occurred during the liquid culture of strains Z-0119 and Z-0118 as the initial pH increased, achieving maximum values of 249.02 μg/mL and 209.47 μg/mL, respectively, at an initial pH of 6. For strain Z-0090, the extracellular flavonoid concentration peaked at 279.05 μg/mL when the initial pH was 7.0. These findings suggested that a slightly acidic pH environment was more favorable for the production of extracellular flavonoids by *Sanghuangporus* during liquid fermentation.

Inoculum volume influenced the rate at which mycelium attained the stationary phase. As showed in Figure 5C, an inoculum volume of 15% resulted in maximum extracellular flavonoid concentrations of 235.09 μg/mL for strain Z-0090 and 240.65 μg/mL for strain Z-0118. Conversely, an inoculum volume of 10% for strain Z-0119 yielded an extracellular flavonoid concentration of 260.88 μg/mL, significantly surpassing the levels observed under the other two shaking speed conditions. In summary, a larger inoculum volume enhanced the extracellular flavonoid production in the three *Sanghuangporus* strains during liquid fermentation.

### 3.6. Response Surface Experimental Design and Results of Sanghuangporus Strains

#### 3.6.1. Response Surface Experimental Design and Results of Strain Z-0090

The results of the response surface experiment for strain Z-0090 were shown in Table 1. Taking the extracellular flavonoid concentration as the response value, the quadratic polynomial for strain Z-0090 was: Y = 300.5 + 9.07A − 6.89B − 12.97C − 4.08AB − 8.7AC + 2.4BC − 37.06A^2^ − 37.86B^2^ − 39.35C^2^. The variance and statistical significance of the quadratic polynomial equation in the response surface analysis were shown in Table 2. This model was statistically significant (*p* < 0.0001). The model terms (A, B, C, AB, AC, A^2^, B^2^, C^2^) were also statistically significant (*p* < 0.05). The lack-of-fit (F) value was 0.4217, whihc showed no significant difference from the pure error (*p* = 0.748). Therefore, this model could be used to predict the optimal concentration of the liquid fermentation substrate for increasing the extracellular flavonoid concentration of strain Z-0090.

**Figure 6:**
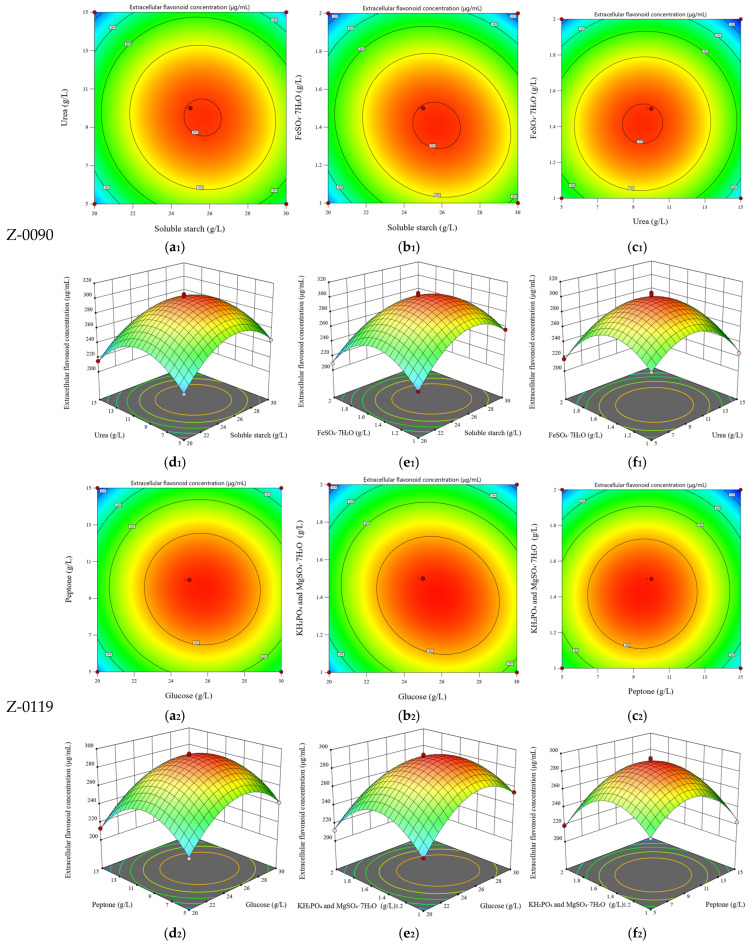

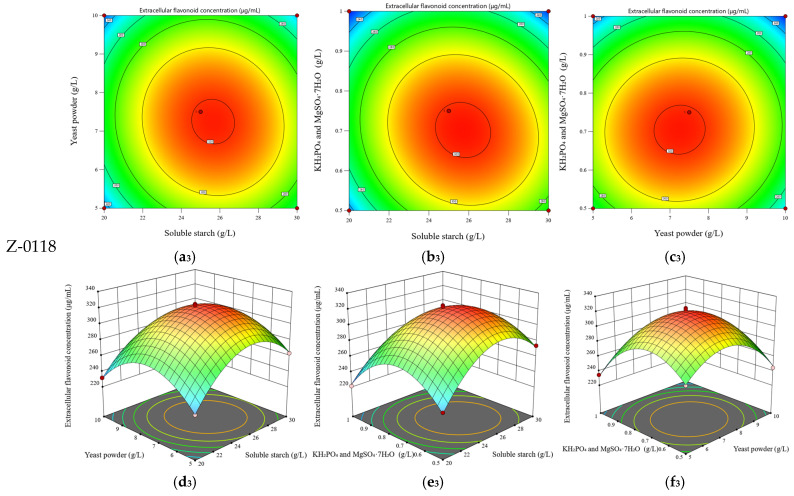
The optimization of the substrate concentration in the liquid culture media of *Sanghuangporus* using the response surface methodology based on the Box–Behnken design. (**a**–**c**) Isoline values of various variables. (**d**–**f**) Interactions between different concentrations of (**d**) carbon sources and nitrogen sources, (**e**) nitrogen sources and inorganic salts, and (**f**) nitrogen sources and inorganic salts in the liquid fermentation medium.

The response surface based on the regression quadratic polynomial equation displayed the interactions among three variables (mass concentration of soluble starch, mass concentration of urea, and mass concentration of FeSO_4_·7H_2_O) and their effects on the extracellular flavonoid concentration (Figure 6a–f). The interactions between the mass concentration of soluble starch and that of urea, as well as between the mass concentration of soluble starch and that of FeSO_4_·7H_2_O, both had significant effects on the extracellular flavonoid concentration of strain Z-0090. The predicted concentrations of each component in the liquid fermentation of strain Z-0090 were 25.75 g/L for soluble starch, 9.48 g/L for urea, and 1.41 g/L for FeSO_4_·7H_2_O. Under these conditions, the extracellular flavonoid concentration of strain Z-0090 was 302.74 μg/mL.

#### 3.6.2. Response Surface Experimental Design and Results of Strain Z-0119

The results of the response surface experiment for strain Z-0119 were summarized in Table 3. Using the extracellular flavonoid concentration as the response variable, the quadratic polynomial for strain Z-0119 was expressed as: Y = 291.68 + 8.80A − 6.51B − 11.09C − 2.09AB − 7.72AC + 2.18BC − 31.78A^2^ − 34.33B^2^ − 34.34C^2^. The variance and statistical significance of this quadratic polynomial equation in the response surface analysis were shown in Table 4. The model demonstrates statistical significance (*p* < 0.0001). In addition, the model terms (A, B, C, AC, A^2^, B^2^, C^2^) were significant (*p* < 0.05). The lack-of-fit (F) value was 1.04, indicating there is no significant difference from the pure error (*p* = 0.4655). Consequently, this model was suitable for predicting the optimal liquid fermentation substrate concentration to enhance the extracellular flavonoid concentration of strain Z-0119.

The response surface derived from the regression quadratic polynomial equation revealed the interactions among three variables of glucose mass concentration, peptone mass concentration, and the mass concentrations of KH_2_PO_4_ and MgSO_4_·7H_2_O, as well as their effects on extracellular flavonoid concentration (Figure 6a–f). Notably, the interaction between glucose mass concentration and the mass concentrations of KH_2_PO_4_ and MgSO_4_·7H_2_O significantly influenced the extracellular flavonoid concentration of strain Z-0119. It was estimated that the optimal concentrations for liquid fermentation of strain Z-0119 were 25.82 g/L for glucose, 9.47 g/L for peptone, and 1.41 g/L for KH_2_PO_4_ and MgSO_4_·7H_2_O. Under these specified conditions, the extracellular flavonoid concentration reached to 293.77 μg/mL.

#### 3.6.3. Response Surface Experimental Design and Results of Strain Z-0118

The results of the response surface experiment for strain Z-0018 were presented in Table 5. Using the extracellular flavonoid concentration as the response variable, the quadratic polynomial for strain Z-0090 was expressed as: Y = 321.01 + 10.07A − 6.95B − 14.42C − 5.00AB − 8.34AC + 3.07BC − 40.78A^2^ − 37.57B^2^ − 40.83C^2^. The variance and statistical significance of this quadratic polynomial equation in the response surface analysis were shown in Table 6. The model demonstrated statistical significance (*p* < 0.0001), and the model terms (A, B, C, AB, AC, A^2^, B^2^, C^2^) were also significant (*p* < 0.05). The lack-of-fit (F) value was 0.4183, indicating no significant difference from the pure error (*p* = 0.7501). Consequently, this model was suitable for predicting the optimal concentration of the liquid fermentation medium to enhance the extracellular flavonoid concentration of Z-0018.

The response surface derived from the regression quadratic polynomial equation displayed the interactions among three variables: the mass concentration of soluble starch, the mass concentration of yeast powder, and the mass concentration of KH_2_PO_4_ and MgSO_4_·7H_2_O, along with their effects on the extracellular flavonoid concentration (Figure 6a–f). Notably, the interactions between the mass concentration of soluble starch and yeast powder, as well as between yeast powder and the mass concentration of KH_2_PO_4_ and MgSO_4_·7H_2_O, significantly influenced the extracellular flavonoid concentration of strain Z-0118. The predicted concentrations for each component in the liquid fermentation of strain Z-0118 were 25.75 g/L for soluble starch, 7.22 g/L for yeast powder, and 0.70 g/L for KH_2_PO_4_ and MgSO_4_·7H_2_O. Under these specified conditions, the extracellular flavonoid concentration of strain Z-0118 reached to 323.57 μg/mL.

### 3.7. Verification Results of the Optimal Substrate Concentration for Sanghuangporus

**Figure 7:**
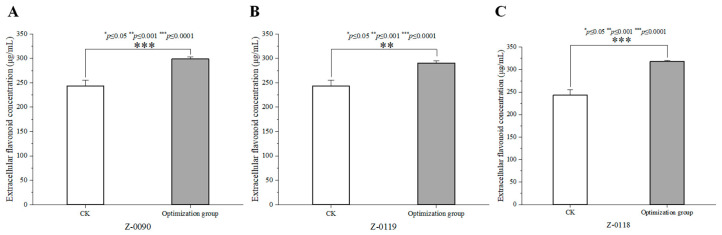
The results of the optimization and verification experiment of the substrate concentration in the liquid fermentation medium. (**A**–**C**) The extracellular flavonoid concentrations of *Sanghuangporus* in the basal medium and the optimized medium.

The extracellular flavonoid concentrations in the liquid fermentation of three strains were determined using the optimized media, with the results displayed in Figure 7. The peak extracellular flavonoid concentrations of these three strains cultured in the basic media which served as the control (refer to Figure 1). Following the optimization, the peak extracellular flavonoid concentration for strain Z-0090 increased to 298.87 μg/mL from the control’s 243.34 μg/mL, marking a 22.82% increase (Figure 7A). Similarly, strain Z-0119 showed a peak concentration of 290.49 μg/mL post-optimization, increase from 256.01 μg/mL in the control, resulting in a 13.47% increase (Figure 7B). For strain Z-0118, the optimized peak concentration reached to 318.03 μg/mL, which reflecting a 16.66% increase compared to 277.33 μg/mL in the control (Figure 7C). These findings confirmed that the prediction model’s effectiveness.

### 3.8. Determination of the Antioxidant Activity of the Fermentation Broth of Sanghuangporus

#### 3.8.1. ABTS Radical Scavenging Activity

**Figure 8:**
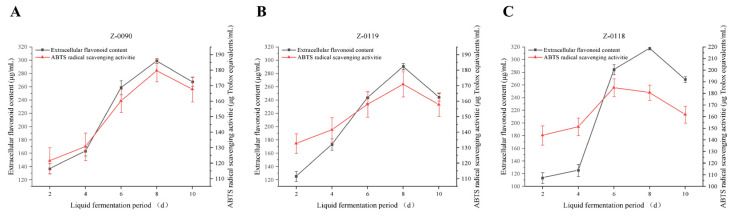
Changes in extracellular flavonoid concentration and ABTS radical scavenging activity of fermentation broth after optimization of liquid fermentation medium. (**A**) Z-0090; (**B**) Z-0119; (**C**) Z-0118.

ABTS can be oxidized by potassium sulfate to produce ABTS radical cations, which are reduced in the presence of antioxidants [12]. This system is highly water-soluble, with a characteristic absorption peak at 734 nm. During the reaction, the solution’s color fades, and the absorbance value decreases significantly as the reduction progresses. Due to its simplicity and stability, this assay method is one of the most widely used for evaluating antioxidant activity [13]. Figure 8 shows the changes in ABTS radical scavenging by the liquid fermentation broth of *Sanghuangporus* over the cultivation period. The scavenging abilities of these three strains exhibited a consistent patterns of increasing steadily in the early cultivation stages and peaking later. As shown in Figure 8C, strain Z-0118 demonstrated the most effective ABTS radical scavenging among these three strains. Its scavenging ability increased from the 2nd to the 6th day of cultivation, which reaching a maximum of 184.96 μg Trolox equivalents/mL on the 8th day.

#### 3.8.2. DPPH Radical Scavenging Activity

**Figure 9:**
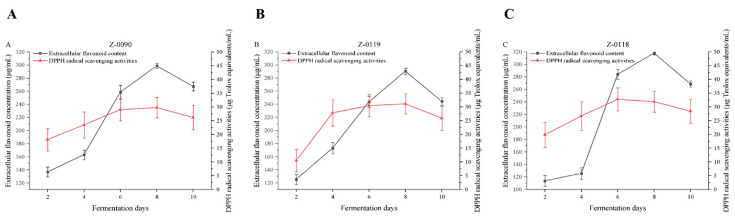
Changes in the concentration of extracellular flavonoids and the DPPH radical scavenging activity of fermentation broth after optimization of the liquid fermentation medium. (**A**) Z-0090; (**B**) Z-0119; (**C**) Z-0118.

DPPH, a synthetic stable free radical, exhibits a strong characteristic absorption peak at a wavelength of 517 nm [14]. When a free radical scavenger is added to the system, the DPPH radicals are reduced, accompanied by a significant change in the color of the solution. Moreover, the reaction system shows good stability. The change in absorbance can be detected using an ultraviolet spectrophotometer, enabling the quantitative analysis of free radical scavenging ability [15]. In this study, the change in DPPH radical scavenging ability during the liquid culture of *Sanghuangporus* was investigated. Strain Z-0118 achieved the highest scavenging ability of 32.75 μg Trolox equivalents/mL. Subsequently, it started to decline slightly from the 8th day but still reached 31.88 μg Trolox equivalents/mL (Figure 9C). According to the experimental results, there was little difference in DPPH scavenging ability among these three strains, which indicating that the *Sanghuangporus* strains in this study possess relatively high antioxidant activity.

#### 3.8.3. Determination of Hydroxyl Radical Scavenging Ability

**Figure 10:**
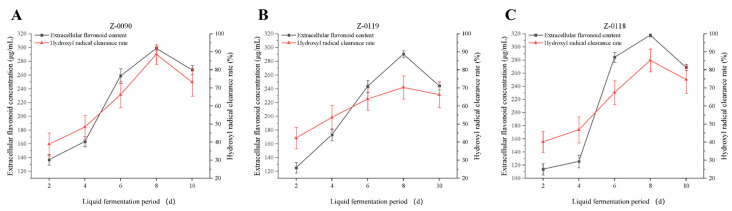
Changes in extracellular flavonoid concentration and hydroxyl radical scavenging ability of fermentation broth after optimization of liquid fermentation medium. (**A**) Z-0090; (**B**) Z-0119; (**C**) Z-0118.

Hydroxyl radicals are among the most potent oxidizing free radicals. Their formation results from the photolysis of nitrates, nitrites, and hydrogen peroxide under natural sunlight, and they also arise from the photochemical reactions of naturally dissolved organic compounds [16]. Notably, the Fenton reaction (Fe^2+^ + H_2_O_2_ → Fe^3+^ + ·OH + OH^−^) and Fenton-like reactions involving transition metals (e.g., Cu^2+^, Mn^2+^) represent key non-photolytic sources of ·OH in both environmental and biological systems; in matrices analogous to fermentation broths, metal ions can form stable complexes with endogenous ligands (e.g., flavonoids, organic acids) to sustain ·OH generation under neutral or slightly acidic conditions, a process closely linked to oxidative stress-mediated biological damage [17]. As a toxic reactive oxygen species in biological systems, hydroxyl radicals can function as effective agents with potential application value while also inducing various pathological reactions and being closely associated with the aging process of organisms [18]. This study investigated the scavenging ability of the *Sanghuangporus* fermentation broth against hydroxyl radicals. The scavenging ability of three strains increased gradually from days 2 to 8, followed by a decrease on the 10th day, which aligned with the trend observed in extracellular flavonoid concentration. Notably, the scavenging rate of strain Z-0090 exhibited consistent changes and was the highest among these three strains, reaching to 88.63% (Figure 10A), thereby indicating that this strain possesses a strong capacity for free radical scavenging.

#### 3.8.4. Total Antioxidant Capacity

**Figure 11:**
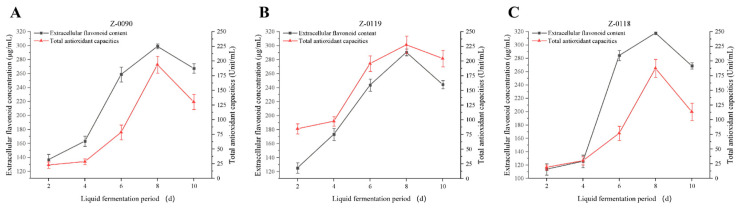
Changes in extracellular flavonoid concentration and total antioxidant capacity of fermentation broth after optimization of liquid fermentation medium. (**A**) Z-0090; (**B**) Z-0119; (**C**) Z-0118.

Reactive oxygen species (ROS), which are continuous by-products of aerobic metabolic processes in both plants and humans, has been shown in previous studies to have a significant physiological value for living systems. This function is not only as essential signaling molecules in numerous biological regulatory processes but also play a critical role in the defense responses of plants against various biotic and abiotic stresses. However, excessive accumulation of ROS can lead to severe cellular damage and toxic effects. Consequently, when ROS levels surpassed the redox regulation capacity and tolerance threshold of organisms, the scavenging of free radicals by antioxidants becomes vital for maintaining normal physiological functions in both humans and plants [19,20,21]. In this study, the trends in total antioxidant capacity and extracellular flavonoid concentration among these three strains exhibited similar patterns, with an increase observed from days 2 to 8, followed by a significant decline beginning on the 8th day (Figure 11).

#### 3.8.5. Superoxide Anion Scavenging Ability

**Figure 12:**
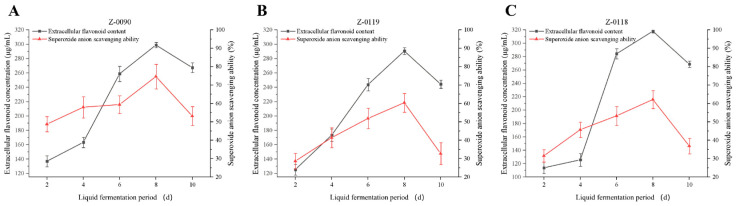
Changes in extracellular flavonoid concentration and superoxide anion scavenging ability of fermentation broth after optimization of liquid fermentation medium. (**A**) Z-0090; (**B**) Z-0119; (**C**) Z-0118.

Superoxide anion radicals are oxygen radicals produced by organisms. As a significant precursor to other reactive oxygen species (ROS), the accumulation of ROS resulting from excessive superoxide generation leads to oxidative damage to DNA, proteins, and other cellular macromolecules. This accumulation has also been linked to the onset and progression of various diseases, including cancer, rheumatoid arthritis, and cardiovascular diseases [22]. In this study, the ability of the fermentation broth to scavenge superoxide anions during the liquid fermentation of *Sanghuangporus* is presented in Figure 12. The scavenging ability peaked on the eighth day for all strains, indicating that, under specific conditions, the superoxide anion scavenging efficiency of these three *Sanghuangporus* strains exhibited a relatively high scavenging rate in the later stages of growth.

### 3.9. Correlation Analysis of Extracellular Flavonoid Concentration and Antioxidant Activity of Fermentation Broth

**Figure 13:**
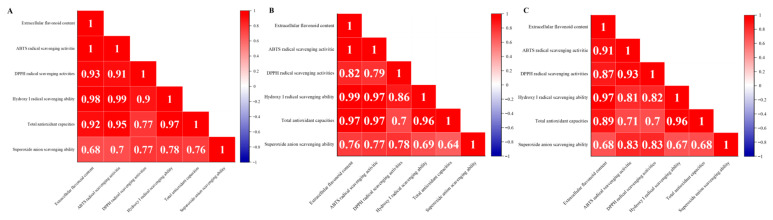
Correlation between extracellular flavonoid concentration in liquid fermentation of *Sanghuangporus* and antioxidant activity of the fermentation broth for strains (**A**) Z-0090, (**B**) Z-0119, (**C**) Z-0118.

As shown in Figure 13A, the extracellular flavonoid concentration of strain Z-0090 exhibited a highly significant positive correlations with ABTS radical scavenging activity and hydroxyl radical scavenging ability. Moreover, it showed a significant positive correlations with DPPH radical scavenging ability and total antioxidant capacity. Notably, the correlation coefficient with ABTS radical scavenging activity attained a maximum value of 1.00, while the correlation coefficient for extracellular flavonoid concentration was lowest in relation to superoxide anion scavenging ability.

As shown in Figure 13B, the extracellular flavonoid concentration of strain Z-0119 was highly positively correlated with ABTS radical scavenging activity, hydroxyl radical scavenging ability, and total antioxidant capacity. Notably, the correlation with hydroxyl radical scavenging ability was the strongest, with a coefficient of 0.99. In contrast, the correlation with superoxide anion scavenging ability was the weakest.

As shown in Figure 13C, the extracellular flavonoid concentration of strain Z-0118 demonstrated a highly significant positive correlation with hydroxyl radical scavenging ability, boasting a correlation coefficient of 0.97. It also showed significant correlations with ABTS radical scavenging activity and total antioxidant capacity, with correlation coefficients of 0.91 and 0.89, respectively. Notably, the superoxide anion scavenging ability had the lowest correlation with the extracellular flavonoid concentration.

## 4. Discussion

The development of bioactive metabolites from the medicinal fungus *Sanghuangporus* is a prominent topic in natural product research. Extracellular flavonoids (EFs), the primary antioxidant components, have garnered significant attention due to their easy extraction from liquid fermentation and notable industrial potential [23]. This study examined three *Sanghuangporus* strains from various sources. Liquid fermentation conditions were systematically optimized using single-factor experiments and the Box–Behnken response surface method. We conducted an in-depth analysis of strain-specific metabolic characteristics and explored the relationship between extracellular flavonoid synthesis and antioxidant activity. This research provides innovative theoretical and technical support for the targeted, high-yield, and precise development of extracellular flavonoids from *Sanghuangporus*.

Carbon sources, nitrogen sources, and inorganic salts are crucial components of the liquid fermentation media, significantly affecting strain growth, metabolism, and the synthesis and accumulation of secondary metabolites like flavonoids. This study revealed notable differences in the optimal selection of these components among *Sanghuangporus vaninii* strain Z-0090 from the wild, *Sanghuangporus vaninii* strain Z-0119 cultivated for many years, and *Sanghuangporus baumii* strain Z-0118. These findings highlighted the specific adaptation mechanisms of *Sanghuangporus* strains to varying nutritional conditions.

Carbon sources are as essential as energy providers for microbial growth and metabolism, as well as precursors for substance synthesis [24]. Strain Z-0090 optimally utilized soluble starch, whereas strain Z-0119 favored glucose. Both strains Z-0090 and Z-0118 preferred soluble starch. This variation may result from the strains’ altered carbon source utilization abilities during domestication [25]. Glucose, being readily available, is quickly absorbed and used by the strains via the glycolytic pathway, fulfilling the energy needs for strain Z-0119′s rapid growth and flavonoid synthesis. This aligns with the metabolic traits of many cultivated *Sanghuangporus*, which favor easily accessible carbon sources [26]. Soluble starch, a slowly available carbon source, requires degradation into glucose by amylase secreted by the strains before utilization. The preference of strains Z-0090 and Z-0118 for soluble starch might be linked to the continuous carbon supply characteristics in their natural habitats. In the wild, the gradual release of starch-like substances, such as plant cell wall polysaccharide degradation products, has driven the evolution of these strains to efficiently degrade and utilize slowly available carbon sources, thus providing a stable carbon skeleton precursor for flavonoid synthesis [27].

The choice of nitrogen sources highlights the metabolic differentiation among the strains. Strain Z-0090 favored the inorganic nitrogen source urea, whereas strains Z-0119 and Z-0118 opted for the organic nitrogen sources peptone and yeast powder, respectively. These organic sources, rich in amino acids, polypeptides, and vitamins, supply essential nitrogen precursors like phenylalanine and tyrosine for flavonoid synthesis, as well as growth factors for these two strains Z-0119 and Z-0118. This satisfied the nutritional requirements of the domesticated strains under artificial culture conditions, aligning with previous findings that “organic nitrogen sources are more conducive to the synthesis of fungal secondary metabolites” [28].

Inorganic salts function as enzyme activators, osmotic pressure regulators, and components of metabolic coenzymes. The optimal combinations of these salts vary which reflecting each strain’s specific mineral requirements. Strain Z-0090 selected FeSO_4_·7H_2_O as its sole inorganic salt, whereas strains Z-0119 and Z-0118 preferred a composite system of MgSO_4_·7H_2_O and KH_2_PO_4_, linked to the enzymatic reaction mechanisms involved in flavonoid synthesis. In the flavonoid biosynthesis pathway, Fe^2+^ serves as the core cofactor for the key enzyme, Fe(II) and 2-oxoglutarate-dependent dioxygenase (Fe/2OG DO) [29]. Mg^2+^ from MgSO_4_·7H_2_O activates hexokinase and phosphofructokinase in glycolysis, while KH_2_PO_4_ supplies phosphorus for nucleic acid synthesis and ATP generation. The combined use of these salts synergistically enhanced the growth and flavonoid synthesis of strains Z-0119 and Z-0118. This finding aligns with the general understanding that “composite inorganic salts are superior to single inorganic salts” in the liquid fermentation of *Sanghuangporus* [30].

The fermentation broths of three *Sanghuangporus* strains demonstrated extensive antioxidant activities, with notable strain-specific differences. Strain Z-0090 exhibited the highest hydroxyl radical scavenging rate at 88.63%, while strain Z-0119 showed relatively balanced antioxidant indices. Strain Z-0118 demonstrated the most effective ABTS radical scavenging ability at 184.96 μg Trolox equivalents/mL. Correlation analysis revealed a correlation coefficient of 1.00 between the extracellular flavonoids (EFs) concentration and ABTS radical scavenging activity for strain Z-0090, 0.99 for strain Z-0119 with hydroxyl radical scavenging ability, and 0.97 for strain Z-0118 with hydroxyl radical scavenging ability. This suggests that EFs are the fundamental material basis for the antioxidant activity of *Sanghuangporus* fermentation broth, which varied with the strength of this association according to the strain [31]. This variation may be related to the proportion of highly active monomers, such as quercetin, in the EFs of each strain [32]. Considering the comprehensive evaluation of yielded extracellular flavonoid and antioxidant activity, strain Z-0118 is recommended for developing high-activity antioxidants, strain Z-0090 is suitable for products targeting hydroxyl radical scavenging, and strain Z-0119 is appropriate for developing broad-spectrum products requiring all antioxidant indices. To become a nutritious supplement, such species must contain beneficial components for the human body, be non-toxic and easily accessible. In the research progress of the past decade, mulberry yellow has fully met these two standards—they are easily available on the market and the flavonoids, polysaccharides, polyphenols and other components it contains all have high nutritional value. In addition, many studies have also proved that mulberry yellow does not contain any toxicity. Based on the above evidence, we have reasons to believe that mulberry yellow can simultaneously supplement the nutrients needed by the human body by regulating multiple physiological pathways, thereby achieving a health care effect. These foreign wines have pointed out a new direction for the research on mulberry yellow as a type of functional food [5].

To contextualize the novelty and superiority of the present study, a systematic comparison of flavonoid yields, fermentation efficiency, and research focus was conducted with previous reports on *Sanghuangporus*. A critical distinction lies in the research object: most prior studies centered on intracellular flavonoids (IFs) from fruiting bodies or mycelia [3,6,30], whereas this work specifically targets extracellular flavonoids (EFs) secreted into the fermentation broth—an understudied fraction with unique advantages of easier extraction, lower purification costs, and industrial scalability [8]. This difference necessitates careful interpretation of yield comparisons, as intracellular flavonoid yields (typically expressed as mg/g dry mycelium) are not directly comparable to extracellular yields (μg/mL broth); thus, we focused on relative efficiency and production feasibility.

In terms of extracellular flavonoid production, the optimized yields of this study (Z-0090: 298.87 μg/mL; Z-0119: 290.49 μg/mL; Z-0118: 318.03 μg/mL) outperformed the few existing reports on *Sanghuangporus* EFs. Zhao et al. [9] optimized submerged fermentation of *Phellinus baumii* (now classified as *Sanghuangporus baumii*) and achieved a total flavonoid yield of 120 μg/mL, but their medium lacked precise inorganic salt modulation and the fermentation period was 14 days—longer than the 8-day peak production period in this study. Gao et al. [22] reported extracellular polysaccharide yields from *Sanghuangporus sanghuang* but only a marginal EF yield of 85.6 μg/mL, likely due to their focus on polysaccharide synthesis rather than flavonoid-targeted medium optimization. The higher yields in our work can be attributed to strain-specific nutrient tailoring: for example, strain Z-0090 (wild *S. vaninii*) preferred urea (inorganic nitrogen) and FeSO_4_·7H_2_O, while strain Z-0119 (cultivar *S. vaninii*) thrived on peptone (organic nitrogen) and a MgSO_4_·7H_2_O-KH_2_PO_4_ composite—an optimization strategy rarely employed in previous studies that typically used uniform medium compositions [27,29].

When comparing with intracellular flavonoid studies (for reference of *Sanghuangporus flavonoid* synthesis potential), Fu et al. [3] extracted 191 mg/g total flavonoids from *Sanghuangporus* mycelia via ultrasound-assisted extraction, but this required energy-intensive post-harvest processing and the actual bioavailable fraction was unclear. Wang et al. [6] obtained 207 mg/g intracellular flavonoids from *S. sanghuang* fruiting bodies, but fruiting body cultivation takes 6–12 months, whereas liquid fermentation produced EFs in 8 days with minimal processing in our study. Even when normalized by culture time, the EF production rate (39.76–41.25 μg/mL/day) in our study is comparable to the intracellular flavonoid accumulation rate (2.7–3.45 mg/g/day) of fruiting bodies, highlighting the efficiency of our EF-focused fermentation system.

Experimental design differences further underscore the contributions of our study. Previous works often used single-factor optimization alone [9,25] or ignored fermentation condition interactions [27], whereas we combined single-factor experiments with Box–Behnken response surface methodology to optimize three key nutrients (carbon/nitrogen sources, inorganic salts) and three culture parameters (inoculum size, rotation speed, pH) simultaneously. This holistic approach addressed the limitations of prior studies where unbalanced nutrients (e.g., excess glucose leading to catabolite repression) or suboptimal oxygen supply (insufficient rotation speed) constrained flavonoid synthesis [26,28]. Additionally, our correlation analysis (r = 0.91–1.00 between EFs and antioxidant activities) provides the direct evidence linking *Sanghuangporus* EFs to ABTS/DPPH/hydroxyl radical scavenging, complementing previous studies that only associated total flavonoids (intra + extra) with antioxidant effects [6,30].

In summary, this study advances existing knowledge by: (1) focusing on the understudied extracellular flavonoids of *Sanghuangporus* with industrial relevance; (2) achieving higher yields and shorter fermentation periods via strain-specific medium/culture optimization; (3) establishing direct correlations between EFs and antioxidant activities; and (4) providing a comparative framework that distinguishes EFs from the more commonly reported intracellular flavonoids. These findings address the bottleneck of low EF yields in previous studies and offer a cost-effective, scalable alternative to fruiting body cultivation or intracellular extraction for flavonoid production.

This study investigated the link between the synthesis of extracellular flavonoids in *Sanghuangporus* and antioxidant activity using systematic strain comparative analysis, which is potential for further studies. Future studies could integrate transcriptomic and metabolomic techniques to identify key genes and metabolic pathways associated with carbon and nitrogen source utilization and the regulation of inorganic salts, thereby elucidating the molecular basis of strain differences. In addition, comparative analyses of liquid and solid-state fermentation should be conducted to assess the compositional differences and yield benefits of extracellular flavonoids under various fermentation conditions, providing a foundation for selecting industrial production methods. This research not only supports the targeted high-yield production of extracellular flavonoids in *Sanghuangporus* but also introduces a novel approach for the refined development and utilization of medicinal fungal strain resources.

## 5. Conclusions

In conclusion, this study focused on optimizing the extracellular flavonoid concentration by adjusting the composition of liquid fermentation medium and fermentation conditions for three *Sanghuangporus* strains, using single-factor experiments and response surface methodology. The optimal conditions for strain Z-0090 included 25.75 g/L of soluble starch, 9.48 g/L of urea, 1.41 g/L of FeSO_4_·7H_2_O, a 15% inoculum volume, a shaking speed of 180 rpm, and an initial pH of 7.0. For strain Z-0119, the optimal conditions were 25.82 g/L of glucose, 9.47 g/L of peptone, 1.41 g/L of KH_2_PO_4_ and MgSO_4_·7H_2_O, a 10% inoculum volume, a shaking speed of 150 rpm, and an initial pH of 6.0. For strain Z-0118, the ideal conditions were 25.75 g/L of soluble starch, 7.22 g/L of yeast powder, 0.7 g/L of KH_2_PO_4_ and MgSO_4_·7H_2_O, a 15% inoculum volume, a shaking speed of 150 rpm, and an initial pH of 6.0. After optimization, the extracellular flavonoid concentration increased by 22.82% in strain Z-0090, 13.47% in strain Z-0119, and 16.66% in strain Z-0118 compared to the control group. The antioxidant activity results of the fermentation broth indicated that strain Z-0090 exhibited the highest hydroxyl radical scavenging rate at 88.63%. Meanwhile, strain Z-0119 showed relatively balanced antioxidant indicators, and strain Z-0118 demonstrated the best ABTS radical scavenging ability at 184.96 μg Trolox equivalents/mL, closely linked to its extracellular flavonoid concentration. These findings suggest significant potential applications in the food industry. For example purified extracellular flavonoids could be added to beverages as natural antioxidants to delay nutrient oxidation and extend shelf life. In addition, they can be incorporated into food products to enhance antioxidant and health-promoting properties, offering a novel pathway for advancing the health aspects of the food industry.

## Figures and Tables

**Table 1:** Response surface design and test results of strain Z-0090.

No.	A: Soluble Starch (g/L)	B: Urea (g/L)	C: FeSO_4_·H_2_O (g/L)	Extracellular Flavonoid Concentration (μg/mL)
1	30	10	2	210.95
2	20	15	1.5	215.06
3	25	10	1.5	297.97
4	25	5	1	245.21
5	25	10	1.5	305.81
6	20	5	1.5	219.11
7	30	10	1	256.51
8	25	5	2	216.71
9	25	10	1.5	297.6
10	25	10	1.5	298.08
11	20	10	2	209.06
12	30	5	1.5	244.26
13	20	10	1	219.82
14	30	15	1.5	223.9
15	25	10	1.5	303.04
16	25	15	2	206.17
17	25	15	1	225.07

**Table 2:** Variance analysis and significance tests of strain Z-0090.

Source	Sum of Squares	Df	Mean Squares	F-Value	*p*-Value
Model	23,268.55	9	2585.39	248.57	<0.0001
A	658.3	1	658.3	63.29	<0.0001
B	379.36	1	379.36	36.47	0.0005
C	1344.73	1	1344.73	129.29	<0.0001
AB	66.5	1	66.5	6.39	0.0393
AC	302.76	1	302.76	29.11	0.001
BC	23.04	1	23.04	2.22	0.1803
A ^2^	5783.31	1	5783.31	556.02	<0.0001
B ^2^	6034.09	1	6034.09	580.13	<0.0001
C ^2^	6520.92	1	6520.92	626.94	<0.0001
Residual	72.81	7	10.4		
Lack of fit	17.49	3	5.83	0.4217	0.748
Pure error	55.31	4	13.83		
Cor Total	23,341.36	16			

R^2^ = 0.9969; adjusted R^2^ = 0.9929; predicted R^2^ = 0.9843.

**Table 3:** Response surface design and test results of strain Z-0119.

No.	A: Glucose (g/L)	B: Peptone (g/L)	C: KH_2_PO_4_ and MgSO_4_·7H_2_O (g/L)	Extracellular Flavonoid Concentration (μg/mL)
1	25	10	1.5	291.58
2	20	10	1	221.79
3	25	10	1.5	290.02
4	30	10	2	213.91
5	30	10	1	254.47
6	30	15	1.5	226.86
7	25	10	1.5	293.94
8	20	10	2	212.09
9	25	15	1	223.02
10	25	10	1.5	294.85
11	25	5	1	242.24
12	30	5	1.5	242.23
13	20	15	1.5	213.08
14	25	5	2	218.65
15	25	15	2	208.15
16	20	5	1.5	220.1
17	25	10	1.5	288.03

**Table 4:** Variance analysis and significance tests of strain Z-0119.

Source	Sum of Squares	Df	Mean Squares	F-Value	*p*-Value
Model	18,063.39	9	2007.04	252.62	<0.0001
A	619.70	1	619.70	78.00	<0.0001
B	339.43	1	339.43	42.72	0.0003
C	983.90	1	983.90	123.84	<0.0001
AB	17.43	1	17.43	2.19	0.1821
AC	238.08	1	238.08	29.97	0.0009
BC	19.01	1	19.01	2.39	0.1658
A^2^	4253.37	1	4253.37	535.36	<0.0001
B^2^	4963.25	1	4963.25	624.71	<0.0001
C^2^	4963.97	1	4963.97	624.80	<0.0001
Residual	55.61	7	7.94		
Lack of fit	24.37	3	8.12	1.04	0.4655
Pure error	31.24	4	7.81		
Cor Total	18,119.01	16			

R^2^ = 0.9969; adjusted R^2^ = 0.9930; predicted R^2^ = 0.9758.

**Table 5:** Response surface design and test results of strain Z-0118.

No.	A: Soluble Starch (g/L)	B: Yeast Powder (g/L)	C: KH_2_PO_4_ and MgSO_4_·7H_2_O (g/L)	Extracellular Flavonoid Concentration (μg/mL)
1	25	10	1	225.23
2	25	5	0.5	266.15
3	25	7.5	0.75	323.25
4	20	10	0.75	231.71
5	30	7.5	0.5	274.22
6	25	7.5	0.75	324.27
7	25	5	1	234.55
8	20	7.5	0.5	236.8
9	30	7.5	1	225.32
10	30	10	0.75	241.26
11	25	7.5	0.75	313.43
12	20	5	0.75	234.06
13	25	10	0.5	244.54
14	25	7.5	0.75	324.81
15	20	7.5	1	221.27
16	30	5	0.75	263.62
17	25	7.5	0.75	319.3

**Table 6:** Variance analysis and significance tests of strain Z-0118.

Source	Sum of Squares	Df	Mean Squares	F-Value	*p*-Value
Model	25,585.11	9	2842.79	167.44	<0.0001
A	811.64	1	811.64	47.81	0.0002
B	386.98	1	386.98	22.79	0.0020
C	1662.91	1	1662.91	97.94	<0.0001
AB	100.10	1	100.10	5.90	0.0455
AC	278.39	1	278.39	16.40	0.0049
BC	37.76	1	37.76	2.22	0.1795
A^2^	7002.91	1	7002.91	412.47	<0.0001
B^2^	5942.31	1	5942.31	350.00	<0.0001
C^2^	7018.38	1	7018.38	413.38	<0.0001
Residual	118.85	7	16.98		
Lack of fit	28.38	3	9.46	0.4183	0.7501
Pure error	90.47	4	22.62		
Cor Total	25,703.95	16			

R^2^ = 0.9954; adjusted R^2^ = 0.9894; predicted R^2^ = 0.9768.

## Data Availability

The original contributions presented in this study are included in the article. Further inquiries can be directed to the corresponding authors.
